# The 2022 nobel prize in physiology or medicine awarded for the decoding of the complete ancient human genome

**DOI:** 10.1016/j.bj.2023.02.004

**Published:** 2023-02-14

**Authors:** Albert Min-Shan Ko

**Affiliations:** a Department and Graduate Institute of Biomedical Sciences, Chang Gung University, Taoyuan, Taiwan; b Cardiovascular Department, Chang Gung Memorial Hospital, Taoyuan, Taiwan; c Healthy Aging Research Center, Chang Gung University, Taoyuan, Taiwan

**Keywords:** 2022 nobel prize in physiology or medicine, Svante pääbo, Ancient DNA, Archaic humans

## Abstract

Since the publication of the first ancient DNA sequence in 1984, experimental methods used to recover ancient DNA have advanced greatly, illuminating previously unknown branches of the human family tree and opening up several promising new avenues for future studies of human evolution. The 2022 Nobel Prize in Physiology or Medicine was awarded to Svante Pääbo, director of the Max Planck Institute for Evolutionary Anthropology in Leipzig, Germany, for his work on ancient DNA and human evolution. On his first day back at work, he was thrown in the pond as part of his institute's tradition of celebrating award winners.

Understanding where we came from is fundamental to appreciating the unique qualities that make up our species. The fossil record has been crucial in piecing together human history up to this point, but it is insufficient for estimating the extent to which early human societies mixed genetically. The ancient DNA experiments that Pääbo developed greatly aided our efforts to piece together the human evolutionary narrative. Pääbo has shown that DNA can be extracted from fossils and then sequenced using next-generation sequencing to reconstruct a high-quality genome [1]. One must be aware that postmortem DNA is typically of low amount and it undergoes fragmentation and chemical damage (such as cytosine deamination, a type of hydrolytic degradation) that can lead to sequencing mistakes due to erroneous nucleotide incorporation [2]. To add to the problem, it may be difficult for scientists to ascertain whether the DNA they uncover in bones is authentic or has been corrupted by microorganisms or human manipulation of fossils. To avoid cross-contamination between samples, this process must be carried out in a specially designed clean lab. The process of recovering ancient DNA is like trying to put together a complex jigsaw puzzle with many of the pieces missing or damaged. DNA sequences, before any kind of analysis can begin, must be cleaned up of various kinds of errors, both those that occur naturally and those that are the consequence of artificial processes.

Due to Pääbo's pioneering work, over the past 40 years, the field of ancient DNA studies has been transformed by the rapid development of both wet and dry laboratory procedures that accommodate varied sample types and stages of preservation [2]. There has been an explosion in the collection of ancient DNA from a wide range of sources, including mammoths older than a million years [3], hair shafts [4], earth sediments [5], early domesticated crops and animal breeds [6,7], bacteria from dental plaque [8], coprolites [9], and the osseous bones of Black Death victims from 1347 to 1348 [10], each providing a unique lens through which to examine human history. As a result, paleogenomics has emerged as a distinct field of research. However, ancient DNA also introduced new problems that must be addressed and remedied, such as ethical concerns, the use of problematic terminology, and the careless equating of different cultural and political categories [2]. Furthermore, ancient DNA/population genetics have been criticized as story-telling when seen in isolation from the relevant archaeological context [11]. Whenever a particular fossil is either unavailable or has yet to be discovered, for instance, it is necessary to draw upon indirect evidence from a number of other places. Past attempts to reconstruct the events of the Pacific colonization without using the ancient DNA from the bones of the Pacific's early settlers is one such example. Helicobacter, a bacterium often found in the stomachs of modern Pacific Islanders, has instead been the focus of genetic variation studies [12].

Pääbo's group was the first to publish the Neanderthal genome draft sequence [13], marking a significant step toward proving that our ancestors had interacted with and reproduced with other branches of the human family tree. To date, the complete Neanderthal genome has been sequenced, and the quality is on par with that of the modern human genome. For his work decoding the genomes of long-extinct archaic humans like Europe's Neanderthals and Asia's Denisovans, Pääbo was awarded the Nobel Prize [14]. The sequencing of the modern human genome has not yet received the same level of Nobel Prize recognition as the ancient human genome. This is an interesting development that warrants our continued attention. The ancient human genome has profound implications for our knowledge of human evolution since it clarifies the relationship between our own species (anatomically modern humans presumed to have developed in Africa around 300,000 years ago) and our older cousins (archaic humans estimated to have emerged from Africa about 1 million years ago). Pääbo compared the genomes of modern and archaic humans and used mathematical models to infer some missing information about our evolutionary history, that when our ancestors migrated out of Africa and into Eurasia some 70,000 years ago, they interbred with archaic humans who had been living in isolation in different parts of the continent for the previous 400,000 years [13,15]. Around 30,000 years ago, these archaic humans became extinct. Possibly, our ancestors assimilated these people into their expanding society. Because of our genetic ties to archaic humans, some present-day human populations have preserved a greater number of ancient human genetic variations than others. Neanderthal DNA makes up 1–2% of the genomes of Europeans and Asians, whereas Denisovan DNA accounts for as much as 6% of the genomes of certain Southeast Asians and Oceanians [13,14,16].

It is a topic of intense research to determine which ancient human genes have benefitted present-day humans and which have not, and what effects this has had on our evolutionary trajectory. In this respect, several cases have been explored by Pääbo. The study of the *FOXP2* gene, for instance, to determine if Neanderthals spoke a language, which might provide insight on how languages emerge [17]. The *SCN9A* gene study looked at whether or not Neanderthals had heightened pain perception to help the body avoid injury and adapt to its environment [18]. The Neanderthal progesterone receptor, which has increased sensitivity to progesterone and provided protection against preterm births, may help explain the factors that led to the extinction of the Neanderthals [19]. There is evidence that Neandertal ancestry increases susceptibility to biliary cirrhosis, Crohn's disease, type 2 diabetes, and severe COVID-19 infection [20,21]. Recent studies of Neanderthal brain development have been conducted to better comprehend their level of intellect [22]. In conclusion, furthering our knowledge of how our ancestors' genes impact our susceptibility to disease might have far-reaching effects on the future of medical treatment.
